# Recycling performance of graphene oxide-chitosan hybrid hydrogels for removal of cationic and anionic dyes

**DOI:** 10.1186/s40580-019-0215-0

**Published:** 2020-02-10

**Authors:** Thi Sinh Vo, Tran Thi Bich Chau Vo, Ji Won Suk, Kyunghoon Kim

**Affiliations:** 1 School of Mechanical Engineering, Sungkyunkwan University, Suwon, 16419 Republic of Korea; 2 Department of Industrial Management, Can Tho University, Can Tho, Vietnam

**Keywords:** Graphene oxide, Chitosan, Composite hydrogel, Dye removal, Filtration, Wastewater treatment

## Abstract

Water is one of the most important resources for human survival and development. Efficient wastewater treatment techniques such as coagulation, filtration, ozonation, and reverse osmosis have been studied to remove toxic materials from water. Implementation of adsorption columns has been proven to be an efficient wastewater treatment method, particularly for the removal of organic contaminants. In this study, we present the preparation of an eco-friendly graphene oxide–chitosan (GC) composite hydrogel column (GCCHC) and its application as a broad-spectrum adsorbent for wastewater treatment. The GCCHC shows a high removal capacity towards different contaminants including both cationic dyes [methylene blue (MB) and rhodamine B (RhB)] and anionic dyes [methylene orange (MO) and congo red (CR)]. Moreover, the samples can be regenerated and recycled without loss of contaminant removal capacity over successive adsorption and washing cycles.

## Introduction

With the rapid economic and industrial developments, water pollution is a rising environmental threat due to the disposal of large amounts of dye-bearing wastewater. Therefore, dye removal from wastewater has received considerable attention with several adsorbents and classes of dyes being investigated. The adsorption of pollutants onto suitable substrates has been recognized as one of the most promising approaches for wastewater treatment owing to its versatility and relatively low cost [1, 2]. Nowadays, several types of materials including polymeric materials, carbon-based materials and silica gel are used as adsorbents for wastewater treatment [3–6]. In addition, new classes of carbon materials have also been exploited as high capacity adsorbents for wastewater treatment [7–12]. Most of these materials exhibit high removal capacity towards a certain type of pollutant but are less efficient toward other types. For example, unmodified carbon nanotubes (CNTs) display high adsorption capacities toward many organic compounds [13, 14], whereas their adsorption capacity toward metal ions is low [15]. In the case of oxidized CNTs or chemically activated carbon (AC), their resulting adsorbents exhibit a low affinity for negatively charged (anionic) dyes [16] due to the oxygen containing functional groups on their surface [17]. Thus, the development of a broad-spectrum adsorbent with high adsorption capacity for wastewater treatment is still a significant challenge.

Graphene, known for its numerous applications [18–20], is a high-performance adsorbent due to its extremely large specific surface area (2630 m^2^ g^−1^) [21]. For the adsorption capacity of graphene, it can be significantly increased when graphene is decorated with functional groups or other materials [22]. Graphene oxide (GO, see Fig. 2), a modified graphene material, contains various oxygen containing functional groups, such as epoxides, hydroxyls, and carboxyls in its basal plane or at edges [23]. Specifically, these oxygen groups can bind to positively charged organic compounds via electrostatic interactions [24]. However, the contribution of GO to dye adsorption can be attributed to the π–π stacking interactions between the aromatic ring of the dye and the basal planes of GO [25]. As observed with other negatively charged adsorbents, GO exhibits a low affinity for anionic dyes due to the strong electrostatic repulsion observed between them [26].

Chitosan (CTS, see Fig. 2) has been recently studied and applied in water treatment [27–29] and is regarded as a green adsorbent due to its excellent biocompatibility and biodegradability [30–33]. Besides, its adsorption capacity is not broad; the amine and hydroxyl groups of CTS act as active sites to trap anionic pollutions, while its effectiveness towards cationic dyes is rather low due to their adverse electrostatic interactions [34]. However, when evaluated as a composite with GO [35], CTS based material achieved rejections of at least 95% for cationic methylene blue (MB), with the mass balances obtained from measurements of the feed, concentrate, and permeate. The dominant mechanism of removal was the physical rejection for both GO particle sizes. On the other hand, Qi et al. prepared GC sponge-based adsorbent with CTS content of 9% had high adsorption capacity (275.5 mg g^−1^) for MB [36]. For the GC (10:1, wt/wt) hydrogel, the maximal adsorption capacities towards cationic MB and anionic Eosin Y were both higher than 300 mg g^−1^ [37]. Also the concept of broad-spectrum adsorbents has received a great deal of attention in recent years. However, the development of adsorbent materials that can simultaneously remove cationic and anionic pollutants from wastewater appears to be a significant challenge. Thus, the combination of GO and CTS to fabricate a broad-spectrum adsorbent may be a promising strategy for wastewater treatment.

Application of an adsorption column is a promising method for removal of various pollutants from wastewater, owing to low cost of adsorbents, simplicity of preparation, and high removal efficiency. However, recyclability of the adsorption bed is an important factor both economically and environmentally. The most common and simplest method for adsorbent regeneration is solvent washing. For example, Fan et al. [38] and Liu et al. [39] prepared magnetic β-cyclodextrin–CTS/GO and β-cyclodextrin/poly(acrylic acid) (PAA)/GO nanocomposites respectively, obtained reasonably good desorption performance with ethanol. Besides, Wu et al. [40] used of a methanol/acetic acid mixture (10:1, wt/wt) achieved good desorption results for rhamnolipid-functionalized GO. But Zhang et al. [41] presented that only 30% of adsorbed MB was desorbed by methanol and that mixing acetic acid by up to 10% (v/v) only elevated the desorption efficiency to about 37% for GO. In addition, desorption of adsorbed MB with the desorption reagents HCl and NaOH solutions were also used with good desorption efficiency [42, 43]. For GC sponge-based adsorbent of Qi et al. [36], resulting indicate that only 30.9% and 29.1% of adsorbed MB were desorbed by ethanol and 0.5 M HCl solution, respectively. However, use of 0.5 M NaOH successfully desorbed MB for four cycles, in agreement with previous studies [42, 43].

Herein, CTS was chosen as a crosslinking agent, which forms strong electrostatic interactions with GO nanosheets. CTS is considered a green adsorbent [33] that can remove anionic dyes via electrostatic interaction [34, 44]. Thus, the incorporation of CTS can provide a GCCHC with the ability of adsorbing anionic dyes. Our results demonstrate that the resulting GCCHCs display a large dye removal capacity towards both cationic dyes (MB and RhB) and anionic dyes (MO and CR). Furthermore, the dye removal capacities of the composite hydrogels toward different adsorbates can be adjusted by changing their composition. Especially, the GCCHCs can be easily recovered by solvent washing (ethanol) after the filtration-adsorption process.

## Experimental

A commercially available GO solution (5 g L^−1^) was obtained from Grapheneall Co. Ltd. (Korea). CTS (degree of deacetylation = 75–85%, medium molecular weight), MB, RhB, MO, CR, and acetic acid (90%, HAc) were purchased from Sigma Aldrich. Syringes (volume capacity = 10 mL and diameter = 1.5 cm) were purchased from DAIHAN Scientific. All chemicals were used as received without any further treatment. The water used in all the experiments was obtained using a Milli-Q ultrapure water treatment system.

**Fig. 1:**
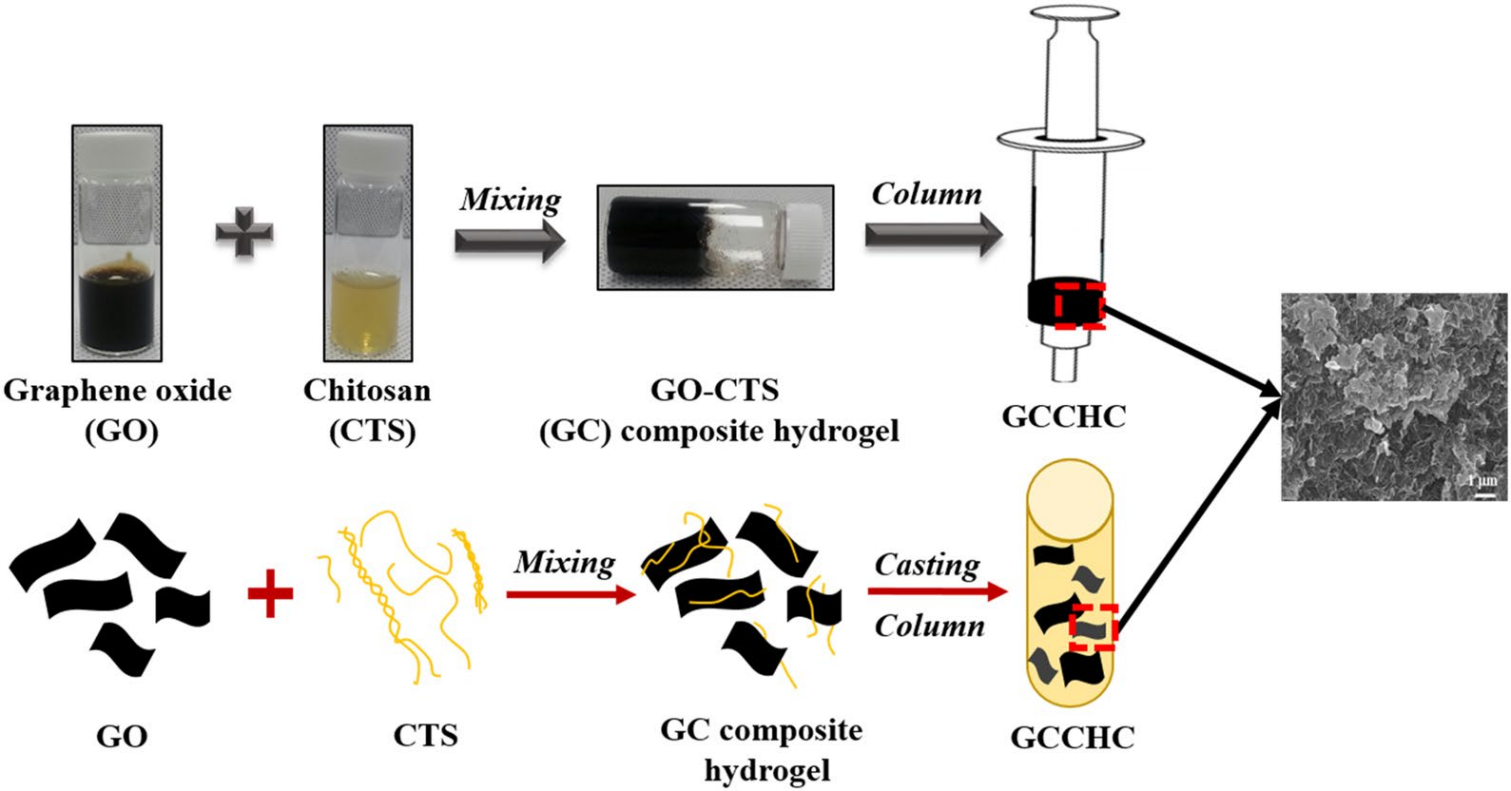
The preparation process of GCCHC

A 2.5 wt% stock solution of CTS was prepared by dissolving 1 g of CTS in 100 mL of 2.5% (v/v) aqueous HAc with stirring overnight. Various amounts of GO suspension were added to 0.25 mL of CTS (5 mg L^−1^) and the resulting mixtures were shaken violently for 10 s to form their hydrogels. Hydrogel formation was confirmed using a tube inversion method. To complete the homogeneous gelation process, the composite hydrogels were subjected to sonication for 10 min. In the sonication, the vial is held on a metal rack and the mixture solution is centrifuged at 4000 rpm for 10 min, at room temperature. The final composite hydrogels contained 5 mg mL^−1^ CTS and various amounts of GO, and were placed in a syringe (volume capacity = 10 mL, diameter = 1.5 cm) to prepare the GCCHCs. The process used to prepare the GCCHC is shown in Fig. 1.

**Fig. 2:**
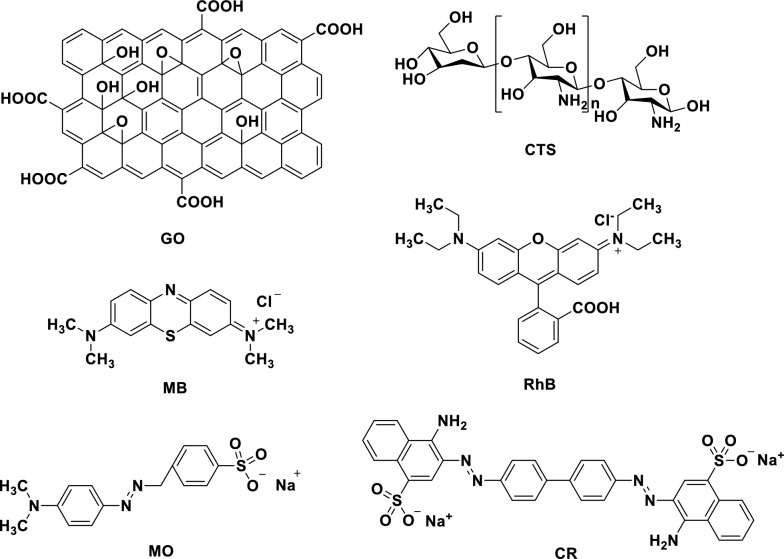
The chemical structures of GO, CTS, MB, RhB, MO, and CR

Cationic (MB and RhB) and anionic (MO and CR) dyes were chosen as the model compounds used in this study (see Fig. 2). Stock solutions were prepared by directly dissolving a known weight of the model compounds in deionized water. The absorbance of the dye solutions was measured using UV–Vis absorbance spectroscopy (SpectraMax M5). Different absorbance wavelengths (660 nm for MB, 550 nm for RhB, 500 nm for CR, and 460 nm for MO) were utilized to determine the absorbance of the residual dye in the resulting solutions.

**Fig. 3:**
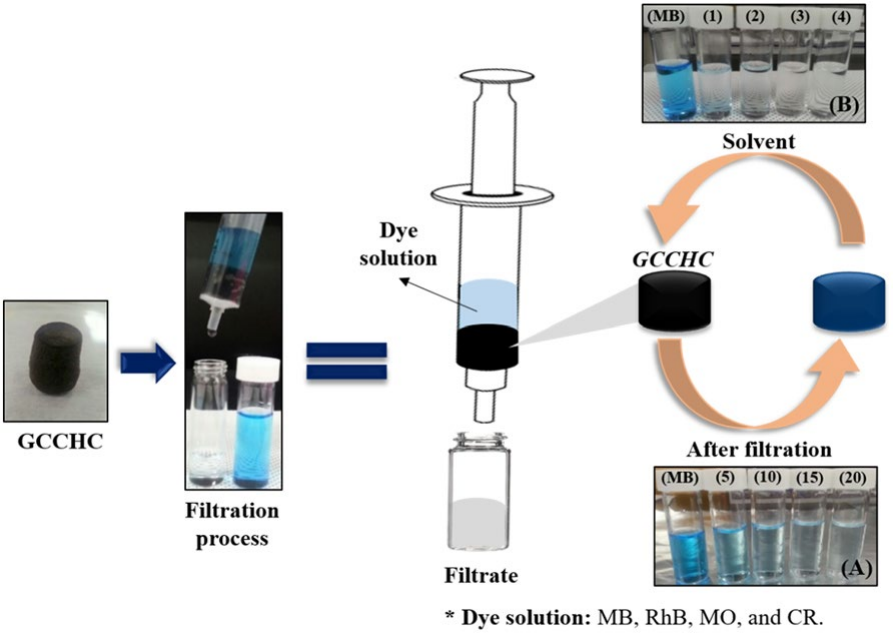
The filtration–adsorption process using the GCCHC

For the adsorption capacities of the GCCHCs, filtration–adsorption experiments were conducted by adding 5 mL of the pollutant solution containing the model dyes (MB, RhB, MO and CR) into a syringe (volume capacity = 10 mL, diameter = 1.5 cm) containing 4 g of the GCCHC sample (composite hydrogel mass) using two methods: Method (A)—direct filtration and Method (B)—filtration after 24 h of immersion (see Fig. 3). Four composite hydrogels with different compositions were tested: GO–CTS5 (GC_5, 5:1, w/w), GO–CTS10 (GC_10, 10:1, w/w), GO–CTS15 (GC_15, 15:1, w/w) and GO–CTS20 (GC_20, 20:1, w/w). After the filtration process was complete, the UV–Vis absorption spectrum of the filtrate was measured using a UV–Vis spectrometer. The dye removal rate (R, %) was calculated according to the following equation: R = (A_o_ − A_t_)/A_o_ × 100%, where A_o_ is the initial absorbance of the dye solution, and A_t_ is the absorbance of the supernatant liquid collected after the filtration process. All the adsorption experiments were carried out at room temperature, which was measured to be 25 ± 2 °C.

In order to investigate the recycling performance of the GC_15 composite hydrogel column as an adsorbent, MB, RhB, MO and CR solutions (C_o_ = 5 mg L^−1^) were used to perform the adsorption cycling experiment. The GC_15 composite hydrogel column was washed with ethanol after the adsorption step and the regenerated sample re-used in the next dye adsorption process (see Fig. 3).

FT-IR spectroscopy was recorded on an FT-IR spectrophotometer (Nicolet 380, Ietled Co.) using the KBr method. The composite hydrogel columns were freeze-dried and triturated prior to the FT-IR measurement. The FT-IR spectra was scanned in the 4000–600 cm^−1^ wavenumber region. Raman spectroscopy was recorded on an XperRam200 spectrometer (BX41M-LED, Olympus) using a laser wavelength of 405 nm. Wide-angle X-ray diffraction (XRD) analysis was carried out on an X-ray diffractometer (D8 ADVANCE, Bruker Corporation) using Cu Kα radiation (λ = 1.5406) at 40 kV and 100 mA, recorded in the 2*θ* range of 5–70°. For the XRD measurements, each sample was prepared by freeze-drying the composite hydrogel followed by being ground into a fine powder. The cross-section structures of the composite hydrogels were measured using a FESEM JSM-7600F instrument (JEOL). The composite hydrogels were frozen in liquid nitrogen, immediately snapped, and then vacuum-dried. DTG curves for the samples were obtained on a Seiko Exstar6000 instrument (TG/DTA6100, SEICO) over a temperature range of 30–700 °C at a heating rate of 10 °C min^−1^. UV–visible spectroscopy was recorded on a SpectraMaxM5 spectrophotometer at room temperature.

## Results and discussion

**Fig. 4:**
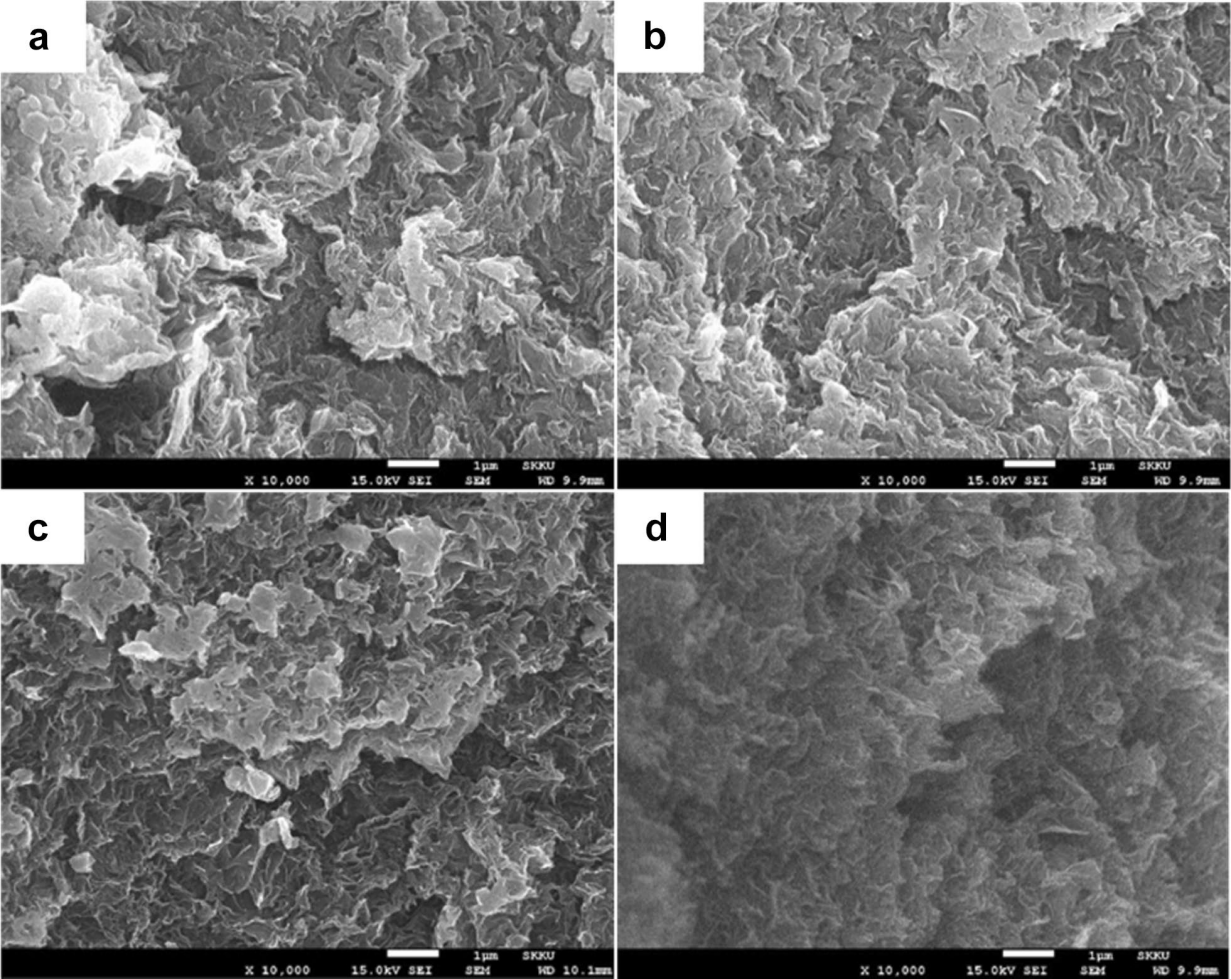
SEM images of the freeze-dried GCCHCs: **a** GC_5; **b** GC_10; **c** GC_15, and **d** GC_20

GO is negatively charged and contains a large number of hydroxyl and epoxide groups, while CTS is a cationic polymer bearing multiple hydroxyl and amino groups, resulting in a complex formation via crosslinking when the two components meet [36, 37, 45]. Specifically, when the GO and CTS solution are mixed and shaken, the resulting mixture immediately lost its fluidity and formed a composite hydrogel via self-assembly between the GO nanosheets and CTS chains. GO nanosheets can be dispersed in water owing to the electrostatic repulsion between each nanosheets, as reported by Kim et al. [46], and any reagents capable of reducing these repulsive forces lead to instability in the GO dispersion. In a concentrated dispersion, Bai et al. showed that large GO nanosheets tend to form a composite hydrogel rather than precipitate when their dispersion becomes unstable because the GO sheets prop each other up before they can reach a compact face-to-face stacking mode [47]. However, CTS is a positively charged polysaccharide due to the presence of amino groups, which can strongly attract the negatively charged GO nanosheets via electrostatic interactions. Moreover, multiple hydrogen bonds can form between the GO sheets and CTS chains to further increase the number of electrostatic interactions. Thus, CTS promotes the formation of the GO composite hydrogel acting as an efficient crosslinking reagent. In addition, the addition of GO can increase the viscosity of the GC solution, which makes the GCCHC stronger after drying [48]. GO presents the sheet-like structure. The SEM images of the GCCHCs (see Fig. 4) show a strong network structure of composite hydrogel, as well as no obvious CTS particles were observed in the SEM images of the GCCHCs, indicating the uniform distribution of the CTS chains on the GO nanosheets. Interconnected pores enable the diffusion of solute throughout the composite hydrogel, which is crucial for further absorption applications. In particular, the self-assembly of these two species takes place immediately when the two solutions come in contact with one another because of the strong electrostatic interactions between GO and CTS, leading to a sharp increase in the viscosity, which is unfavorable for mixing. Therefore, a slight inhomogeneity of the composite hydrogel is inevitable during the shaking process.

**Fig. 5:**
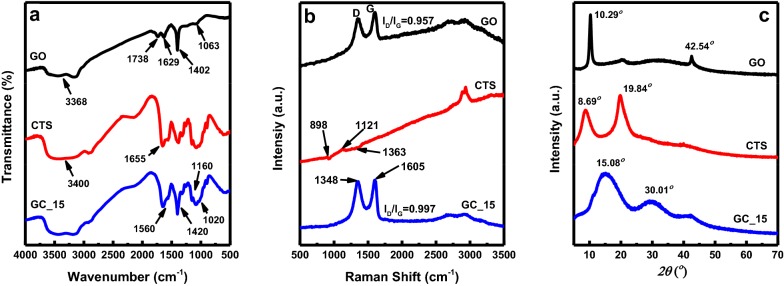
**a** FT-IR, **b** Raman and **c** XRD spectra of GO, CTS, and GC_15

As shown in Fig. 5a, the presence of hydroxyl groups (a strong –OH peak at 3368 cm^−1^), carboxyl groups (C=O at 1738 cm^−1^), epoxy groups (C–O–C at 1402 cm^−1^), and alkoxy groups (C–O at 1063 cm^−1^) are clearly visible, which are in good agreement with that previously reported for GO [49, 50]. The spectrum also shows a C=C peak at 1629 cm^−1^ corresponding to the remaining sp^2^ character. For CTS, the peak at 3400 cm^−1^ corresponds to the N–H stretching vibration, and the absorption peaks at 1020 and 1160 cm^−1^ can be attributed to the primary (C_6_–OH) and secondary (C_3_–OH) alcohol groups, respectively [48, 51]. The peak at 1655 cm^−1^ was assigned to the carbonyl stretching vibration of the acetylated amino groups [48]. Clearly, when compared with pure CTS and GO, both peaks observed at 1655 cm^−1^ belonging to the N–H stretching vibrations of CTS and 1738 cm^−1^ related to the C=O stretching vibrations of GO were significantly decreased in the GCCHC. These results prove that there was an interaction formed between them. In other words, hydrogen bonds and/or electrostatic interactions are formed between the residual oxygen-containing groups in the GO sheets and the –NH_2_ groups on the macromolecular chains of CTS, which has been reported by Travlou et al. [25]. In the Raman spectrum recorded for CTS, the peak observed at 898 cm^−1^ was attributed to NH_2_ wagging (see Fig. 5b). The multiple peaks observed at 1121 cm^−1^ can be attributed to the ether and glycosidic bond stretching vibrations, and the band at 1363 cm^−1^ was associated with methyl group bending vibrations [52]. For the GCCHC, two significant peaks at 1348 and 1605 cm^−1^ were observed, corresponding to the D and G bands of the incorporated GO sheets. The addition of GO leads to the peaks due to CTS films becoming less visible as the intensities of the characteristic D and G bands of GO are greater than those of the CTS bands. The D and G bands show no shift and the I_D_/I_G_ ratio is virtually unchanged in the pristine GO and GCCHC, indicating little or no change in the sp^2^ nature and size of the GO nanosheets occurred [53, 54].

In addition, XRD was used to evaluate the crystallinity of the GCCHC (see Fig. 5c). The XRD pattern of GO shows a sharp peak at 10.29°, corresponding to the interplanar distance between the GO sheets [24]. The XRD pattern of pure CTS powder exhibits two typical peaks at 8.69 and 19.84°, which are attributed to its hydrated (I) and anhydrous (II) crystal forms, respectively [51]. These CTS peaks were not observed in the XRD pattern obtained for the GCCHC, indicating that the CTS chains are well distributed on the GO nanosheets. Compared to GO, The XRD pattern obtained for GCCHC shows peaks at 15.08 and 30.01°. These peaks are significantly broadened, suggesting an increase in the structural disorder.

**Fig. 6:**
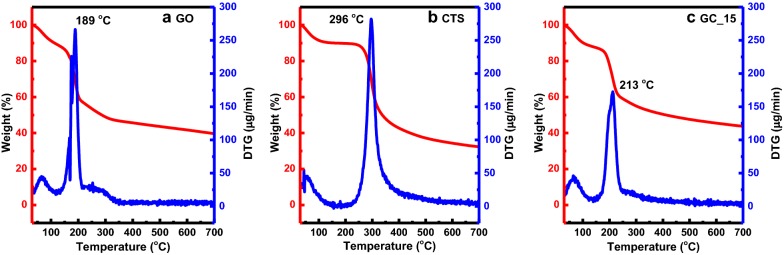
TGA and DTG results obtained for **a** GO, **b** CTS, and **c** GC_15

The thermogravimetric profiles of the as-obtained composite hydrogel materials are illustrated in Fig. 6. The initial weight loss for all the samples at ~ 50 °C reflects the loss of moisture from the samples. The GCCHC demonstrates enhanced thermal stability when compared with GO, which was mainly attributed to the self-assembly and crosslinked structures in the composite hydrogel materials. One obvious mass loss peak is observed at 213 °C for the composite hydrogel materials, which mainly originates from the pyrolysis of the oxygen-containing groups in the GO nanosheets and CTS molecules [55]. Especially, GO-based composite materials have been previously reported to demonstrate various weight retention ratios at a high temperature range, mainly due to the change in the self-assembly of the carbon composites [56–58]. In the study, the as-prepared composite materials clearly exhibit enhanced thermal stability in comparison with pristine GO.

**Fig. 7:**
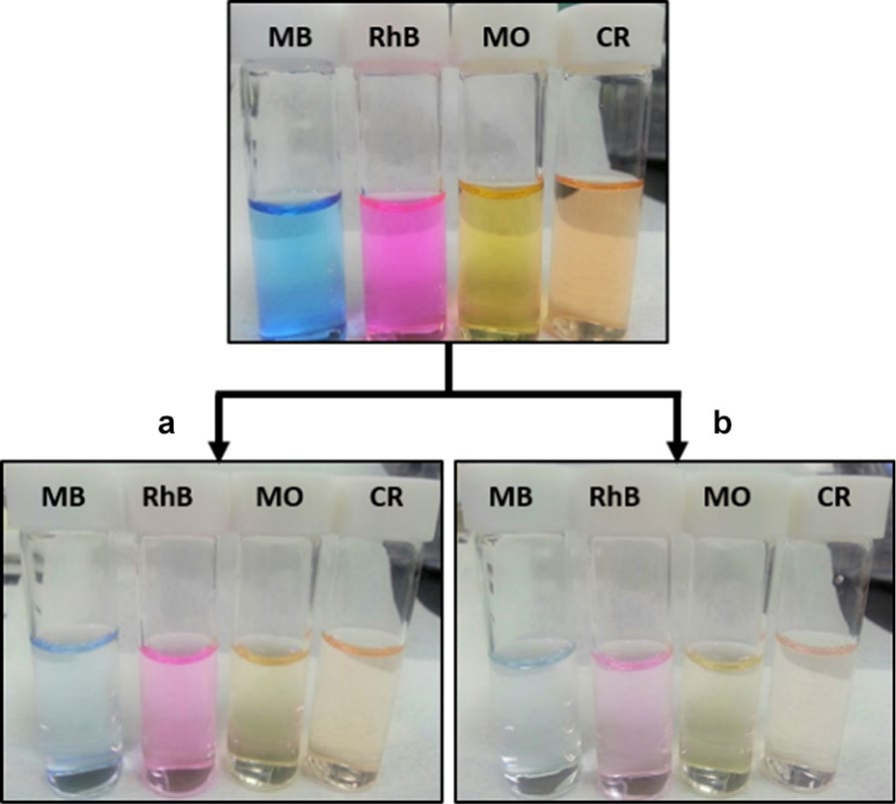
The color of the treated dye solutions using GC_15 composite hydrogel column via **a** direct filtration and **b** filtration after 24 h of immersion

Dye removal from water using an adsorption column is an attractive technique because it allows non-stop operation of a general adsorbent. Here, the GCCH can be used as a packing material to fabricate a column for wastewater treatment. The interconnected channels in the composite hydrogel allow the penetration of water, while the dye molecules are adsorbed by the GCCH. A short column (diameter = 1.5 cm) containing 4 g of the GC_20, GC_15, GC_10 and GC_5 composite hydrogels as column packing was fabricated and used to remove the model dyes from water via a filtration–adsorption process. Four dye solutions with an initial concentration of 5 mg L^−1^ are added onto the column and the filtration–adsorption experiments carried out using two methods: Method (A)—direct filtration and method (B)—filtration after 24 h of immersion. As shown in Fig. 7, the color of the treated samples obtained using the GC_15 composite hydrogel column almost disappeared completely. Namely, method (B) gives a direct visual impression of the fast kinetics observed for MB, RhB, MO, and CR adsorption on the GCCHCs.

**Fig. 8:**
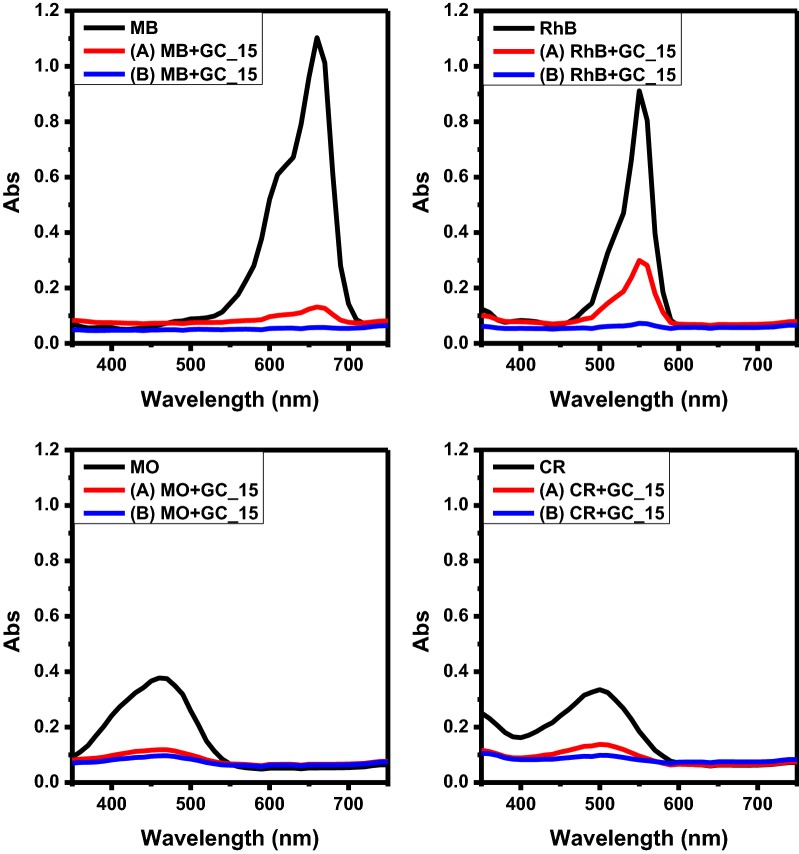
The UV–Vis adsorption spectra recorded for the model dye solutions before and after filtration using the GC_15 composite hydrogel column: **a** direct filtration and **b** filtration after 24 h of immersion

In order to evaluate the adsorption capacity of the GCCHC, the absorbance value of the outlet solution was recorded during the filtration process. After filtration, the absorbance value of the MB, RhB, MO and CR solutions decreased to nearly zero, indicating the complete removal of the dyes, as shown in Fig. 8. Specifically, after filtration, the absorbance value of the MB, RhB, MO and CR solutions obtained using method (B) decrease more than with method (A). This means that the combination of immersion and filtration in method (B) can efficiently remove dye molecules from water, as depicted in Fig. 8. Detailed studies on the dye molecule removal mechanism in method (B) are still underway in our laboratory.

**Fig. 9:**
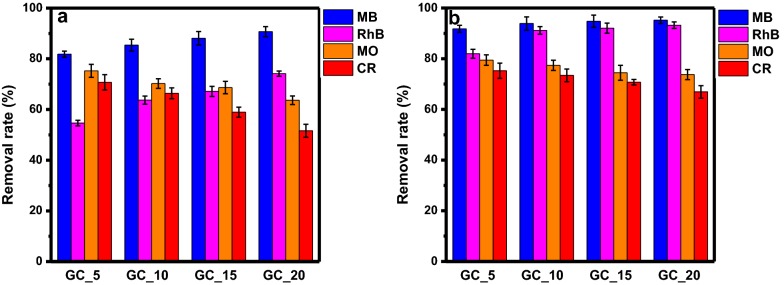
Dye removal rate before and after filtration through the GCCHCs via **a** direct filtration and **b** filtration after 24 h of immersion

For filtration membrane, Abolhassani et al. fabricated GC composite as a film by casting method [35], achieved rejections of at least 95% for cationic methylene blue (MB) during filtration process and suggested that the dominant mechanism of removal is the physical rejection for both GO particle sizes. On the other hand, Qi et al. prepared GC sponge-based adsorbent with CTS content of 9% had high adsorption capacity (275.5 mg g^−1^) for MB during a filtration process [36]. For the GC (10:1, wt/wt) hydrogel, the maximal adsorption capacity towards cationic MB was higher than 300 mg g^−1^ [37]. Here, we prepared GCCHC as a broad-spectrum adsorbent had high removal rate with combination of immersion and filtration. Specifically, the removal rate using method (B) is higher than that of method (A) for each of dye solution [i.e.: 10.00%—GC_5; 8.50%—GC_10; 6.71%—GC_15; 4.48%—GC_20 for MB, 27.32%—GC_5; 27.47%—GC_10; 24.90%—GC_15; 19.10%—GC_20 for RhB, 4.21%—GC_5; 7.16%—GC_10; 5.80%—GC_15; 10.10%—GC_20 for MO, and 4.53%—GC_5; 7.01%—GC_10; 11.78%—GC_15; 15.30%—GC_20 for CR] as shown in Fig. 9. It is indicated the combination of immersion and filtration using the GCCHC was successful during the filtration-adsorption process. In addition, the dye removal capacities of the GCCHCs toward different dyes can be adjusted by changing their composition. For example, GC sponge-based adsorbent with CTS content between 9 and 41% had good filtering performance [36]. The GC sponge with CTS content of 9% had adsorption capacity of 275.5 mg g^−1^ for MB. Both electrostatic attraction and hydrophobic interactions are responsible for MB adsorption by GC sponges [36]. For MB solution with the same initial concentration, the adsorption capacity is about 300 mg g^−1^ with GC (10:1, wt/wt) and GC (20:1, wt/wt) hydrogels as adsorbents, while it dwindles down to 150 mg g^−1^ with GC (5:1, wt/wt) as an adsorbent [37]. Here, the removal rate of MB and RhB increase slowly upon increasing the GO content in the GCCHC, but the removal rate of MO and CR decrease slowly upon increasing the CTS content in the GCCHC (see Fig. 9).

As mentioned above, the GO in GCCHC adsorbs organic dyes mainly via electrostatic attractions, π–π interactions, and hydrogen bonding [59, 60]. However, for hydrogen bonding, the adsorption of MB, RhB, MO, and CR follows a monolayer coverage mechanism, which is contradictory to multilayer adsorption mediated by intermolecular hydrogen bonds between dye molecules [25, 61], so it can be readily ruled out. On the other hand, π–π interactions require high adsorption capabilities toward both cationic and anionic dyes, while electrostatic interactions plays a secondary role in the adsorption of cationic dyes [25, 62]. Thus, the contribution of GO in GCCHC to dye adsorption can be attributed to the π–π dispersion interactions between the aromatic ring of the dye and the basal planes of GO [25]. For CTS in GCCHC, the amine and hydroxyl groups in CTS act as active sites for trapping mainly anionic dyes, while its effectiveness towards cationic dyes is rather low due to their adverse electrostatic interactions [34]. Thus, we believe that GCCHCs containing CTS will be better column packing materials to remove anionic dyes.

**Fig. 10:**
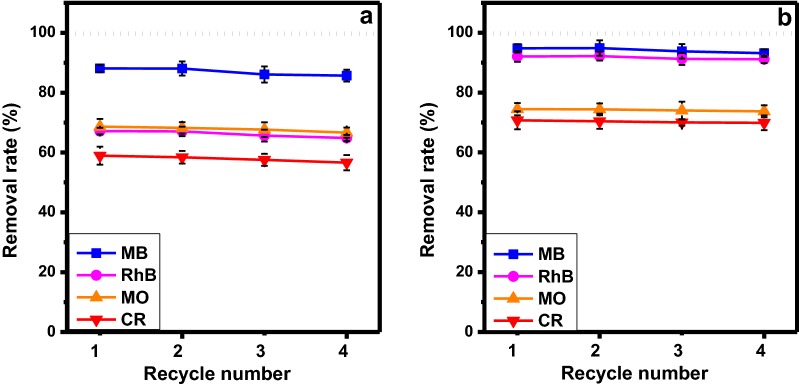
The dye removal rate obtained for the GC_15 composite hydrogel column in the reuse process using **a** direct filtration and **b** filtration after 24 h of immersion

In order to investigate the recycling performance of the GCCHC as an adsorbent, model dye solutions of MB, RhB, MO and CR (C_o_ = 5 mg L^−1^) were used to perform the adsorption cycling experiment. The GC_15 composite hydrogel column was washed with ethanol after adsorbing the MB, RhB, MO and CR solutions and the regenerated samples were used in the next adsorption step. Magnetic β-cyclodextrin–CTS/GO [38] and β-cyclodextrin/PAA/GO nanocomposites [39] obtained reasonably good desorption performance with ethanol. Their adsorption capacities did not change significantly after five cycles. Besides, the adsorption capacities of rhamnolipid-functionalized GO did not also change after five cycles when use of methanol/acetic acid mixture (10:1, wt/wt) [40]. GC sponge-based adsorbent [36], resulting indicate that use of 0.5 M NaOH successfully desorbed MB for four cycles. However, GC hydrogel as a adsorbent [37] had not still been investigated yet for recyling performance. Herein, as shown in Table 1 and Fig. 10, the removal rate using method (B) decreased slightly less than that of method (A) for each of dye solution over 4 cycles [i.e.: 2.37%—MB; 2.30%—RhB; 2.01%—MO; 2.33%—CR in method (A) and 1.60%—MB; 0.94%—RhB; 0.7%—MO; 0.81%—CR in method (B)]. The combination of immersion and filtration using the GC_15 composite hydrogel column was successful during the reuse process, demonstrating that GCCHC is a reusable adsorbent.

**Table 1:** The dye removal rate obtained for the GC_15 composite hydrogel column during the reuse process

Cycle number	Dye removal rate, R (%)							
	Method A				Method B			
	MB	RhB	MO	CR	MB	RhB	MO	CR
1	88.08 ± 1.23	67.15 ± 1.11	68.65 ± 2.56	58.94 ± 2.00	94.79 ± 1.39	92.05 ± 1.78	74.45 ± 2.06	70.72 ± 3.01
2	88.08 ± 2.36	67.05 ± 1.59	68.24 ± 1.89	58.40 ± 2.12	94.87 ± 2.60	92.17 ± 1.49	74.39 ± 1.89	70.40 ± 2.52
3	86.08 ± 2.69	65.65 ± 1.99	67.65 ± 2.45	57.55 ± 1.99	93.78 ± 2.43	91.25 ± 2.00	74.00 ± 2.95	70.02 ± 1.09
4	85.71 ± 1.98	64.85 ± 1.02	66.64 ± 1.69	56.61 ± 2.56	93.19 ± 1.28	91.11 ± 1.27	73.73 ± 1.99	69.91 ± 2.46

## Conclusion

In summary, GCCHCs have been successfully prepared using a self-assembly method and syringe apparatus. In the composite hydrogel materials, GO nanosheets are crosslinked by CTS chains to form a network structure. The structure in the composite hydrogel materials facilitates the diffusion of adsorbates in the composite hydrogels and increases the dye removal capacity of the GCCHC. The dye removal capacity changes with the composition of the composite hydrogel, for example, increasing the GO content leads to a higher dye removal capacity towards cationic dyes (MB and RhB), whereas increasing the CTS content leads to a higher dye removal capacity towards anionic dyes (MO and CR). Therefore, its dye removal capacity depends on changing the content and nature of the each of the reactants to effectively remove both cationic and anionic dyes from water. These results prove that the GCCHC can be used as a broad-spectrum adsorbent for various types of water contaminants. Furthermore, they show that the GCCH can be used as column packing to fabricate a column used to purify water via simple filtrations with a high processing capacity. The dye removal ability remained unaltered over four successive adsorption and washing cycles. Hence, the GCCHC-based broad-spectrum absorbent is a promising candidate for wastewater treatment due to its low cost and the biocompatibility of GCCH.

## Data Availability

The datasets used and/or analysed during the current study are available from the corresponding author on reasonable request.

## Abbreviations

GO: graphene oxide
CTS: chitosan
GC: graphene oxide-chitosan
GCCHC(s): graphene oxide-chitosan composite hydrogel column(s)
GCCH(s): graphene oxide-chitosan composite hydrogel(s)
MB: methylene blue
RhB: rhodamine B
MO: methylene orange
CR: congo red
CNTs: carbon nanotubes
AC: activated carbon
PAA: poly(acrylic acid)
R: dye removal rate
(FE)SEM: (field-emission) scanning electron microscope
FT-IR: Fourier-transform infrared spectroscopy
XRD: X-ray diffraction
TG: thermogravimetry
DTG: derivative thermogravimetry
